# Antioxidant Activity and Profile of Phenolic Compounds in Selected Herbal Plants

**DOI:** 10.1007/s11130-022-00989-w

**Published:** 2022-07-02

**Authors:** Kamil Foss, Katarzyna E. Przybyłowicz, Tomasz Sawicki

**Affiliations:** Department of Human Nutrition, Faculty of Food Sciences, University of Warmia and Mazury in Olsztyn, Słoneczna 45F, 10-719 Olsztyn, Poland

**Keywords:** Herbs, Flavonoids, Phenolic acids, Stilbenes, Bound compounds, Free compounds

## Abstract

Herbs are characterized by a high content of biologically active substances that positively affect human health. Phenolic compounds are one of the main bioactive compounds in these plants with highly beneficial properties (*e.g*., anti-carcinogenic, cardioprotective, immune system support and antibacterial). Therefore, this study aimed to determine the composition of free and bound phenolic compounds and antioxidant activity in 10 different herbs (bogbean leaves, sage leaves, whole Indian hemp, whole heartsease, whole horsetail, whole blessed thistle, whole thyme, chamomile flower, corn silk and pine buds). Phenolic compounds were analyzed using the HPLC-TOF-MS/MS method, total phenolic (TP) and flavonoid (TF) contents were measured using spectrophotometric methods, while antioxidant activity was determined by ABTS and DPPH assays. The highest content of free TP was found in thyme, while sage was characterized by the highest range of these compounds released from ester and glycosidic derivatives by hydrolysis. In turn, the highest values of TF in non-hydrolyzed extracts were found in Indian hemp. The highest values of TF, including bound phenols were observed in extracts obtained from sage leaves. In the analyzed herbs thirty-three phenolic compounds were found, constituting 16 phenolic acids, 9 flavonoids and eight stilbenes. Moreover, the analyzed compounds appeared in the bound form than in the free form. The antioxidant activity of herb extracts differed significantly across varieties (*P* < 0.05). The research showed that each tested herb possesses its own fingerprint of phenolic compounds and antioxidant properties.

**Supplementary Information:**

The online version contains supplementary material available at 10.1007/s11130-022-00989-w.

## Introduction

Herbs are defined as non-wood plants whose stems are not completely lignified. These plants are usually annuals or biennials that die shortly after flowering [1]. In addition to herbaceous plants, this group also includes shrubs, some vegetables and perennials [2]. Herbs are characterized by a high content of biologically active substances which positively affect human health (*e.g.*, essential oils, and phenolic compounds) [3].

As mentioned above, herbs owe their biological activity and influence on the human body to the presence of a number of biologically active substances [4]. The content and, consequently, the effect of biologically active compounds in herbs depends on many factors, such as growing conditions, plant care, herb stabilization, as well as processing and storage [3]. These factors can largely determine the properties of the end product. According to the literature data, the differences in biological activity between products obtained from the same plant may differ up to several thousand times [5].

Phenolic compounds are substances with anti-carcinogenic, cardioprotective, immune system support, antibacterial, antiviral and antifungal properties and protect the skin against UV radiation [6, 7]. Phenolic compounds are synthesized via the shikimic acid and phenylpropanoid acid pathways. Phenolic compounds include flavonoids (*e.g*., anthocyanins, flavones) and phenolic acids (derivatives of cinnamic and benzoic acids). It is worth noting that although these two groups are the main polyphenols, various authors also include lignans, stilbenes and tannins, etc. [8]. More than 8000 phenolic compounds have been identified and described, with half of them being flavonoids [9–11]. These compounds are present in plants in the free form and as ester and/or glycosidic derivatives. However, the health effects of bioactive polyphenols are determined by their bioavailability which is influenced by many factors, including phenolic structure, chemical interactions, food processing and the food matrix components [12, 13]. There is also no information about free and conjugated phenolic compounds in different herbs. Consequently, chemical composition and biological activity of herbs need to be analyzed to determine the functional properties of these herbs after consumption and use in producing plant-fortified functional food. This study aimed to investigate the phenolic compound composition and antioxidant capacity of 10 different herbs, which are not yet commonly consumed but may be of great importance in maintaining and promoting human health and longevity.

## Materials and Methods

The Materials and Methods section is presented as supplementary material.

### Results and Discussion

Phenolic compounds occur in esters with carboxylic acids or glucose. In an acidic environment, these compounds may undergo hydrolysis, which breaks ester and glycosidic bonds, leading to an increase in the number of free compounds [14]. Therefore, the TP and TF contents, individual phenolic compounds, and AA were determined in herbal extracts not subjected to the hydrolysis process after alkaline hydrolysis (phenolic compounds released from ester derivatives) or acid hydrolysis (phenolic compounds released from glycosidic derivatives) to better understand the distribution of phenolic compounds in herbs as well as their potential bioactive properties.

### TP and TF Contents in Selected Herbs

**Fig. 1:**
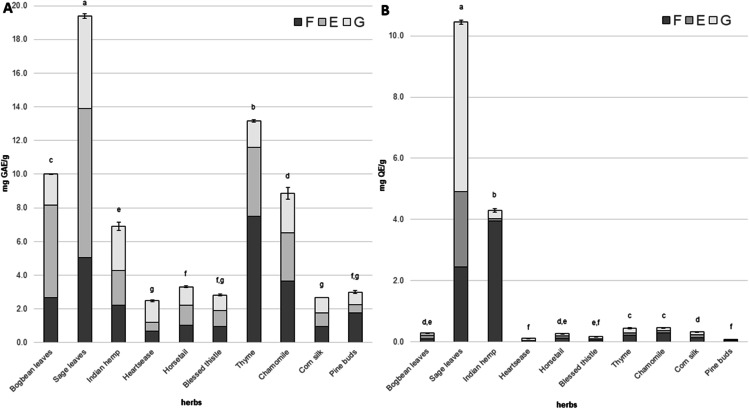
Total phenolic content (TP) and total flavonoid content (TF) in tested herbs. F - free forms of polyphenolic compounds; E - polyphenolic compounds released from ester bonds; G - polyphenolic compounds released from glucoside bonds

The free and conjugated TP and TF contents determined in the extracts of tested herbs are presented in Fig. 1. The TP concentration in herb samples ranged from 2.49 to 19.38 mg GAE/g. The highest concentration of TP compounds was detected in sage leaves (19.38 ± 0.13 mg GAE/g) (Fig. 1a), while the lowest content of TP was noted in heartsease (2.49 ± 0.05 mg GAE/g) and corn silk (2.69 ± 0.00 mg GAE/g). The free form of these compounds was dominant in five herbs and constituted 34.4% (blessed thistle) to 59.0% (pine buds) of the TP concentration. However, TP compounds released from glycosidic derivatives dominated in Indian hemp and heartsease, and their percentage contributions were 37.9 and 52.3%, respectively. For phenolic compounds released from ester derivatives, their percentage contribution to the TP content was the highest in three herbs (horsetail - 35.9%, bogbean leaves - 54.8% and sage leaves - 45.7%).

Significant differences were also found in the analyzed herb samples in terms of TF content (Fig. 1b). The TF content ranged from 0.08 to 10.45 mg QE/g. The highest TF value was found in the sage leaves (10.45 ± 0.07 mg QE/g), while the lowest values were observed in heartsease (0.12 ± 0.00 mg QE/g) and pine buds (0.08 ± 0.00 mg QE/g). Moreover, it was noted that free flavonoids were the dominant compounds in most of the herbs studied. Six herbs (Indian hemp, horsetail, thyme, chamomile, corn silk, and pine buds) had the highest contribution of the free form of flavonoid compounds. Their percentage contribution ranged from 41.79% (corn silk) to 91.9% (Indian hemp) of the TF content. Flavonoid compounds released from ester bonds dominated only in the bogbean leaves (42.5%). However, the flavonoids released from glycosidic bonds were dominant in heartsease (64.0%), sage leaves (53.1%) and blessed thistle (39.2%).

To the best of the authors’ knowledge, only scarce information regarding the TP and TF contents in tested herbs has been reported in the literature. In the available literature, researchers have mainly focused on determining the free form of TP and TF compounds. However, the TP values obtained for the sage are comparable [15, 16], although the data available for horsetail are higher than in the current study [17], and the data available for blessed thistle was lower [18]. In the case of TF content, the available data showed that the concentration of these compounds was almost three times higher than the results presented in the current study [19]. However, the data available for Indian hemp, thyme and blessed thistle are lower [18, 20, 21]. In comparison, the data available for heartsease thistle and horsetail was higher than the data obtained in the current study [17]. The result of such a significant difference in the TP and TF concentrations in herbs may be the influence of the variety, climatic and growing conditions [3]. Moreover, the extraction method is also crucial for the final level of bioactive compounds in the obtained extracts [22, 23].

### Antioxidant Activity

**Fig. 2:**
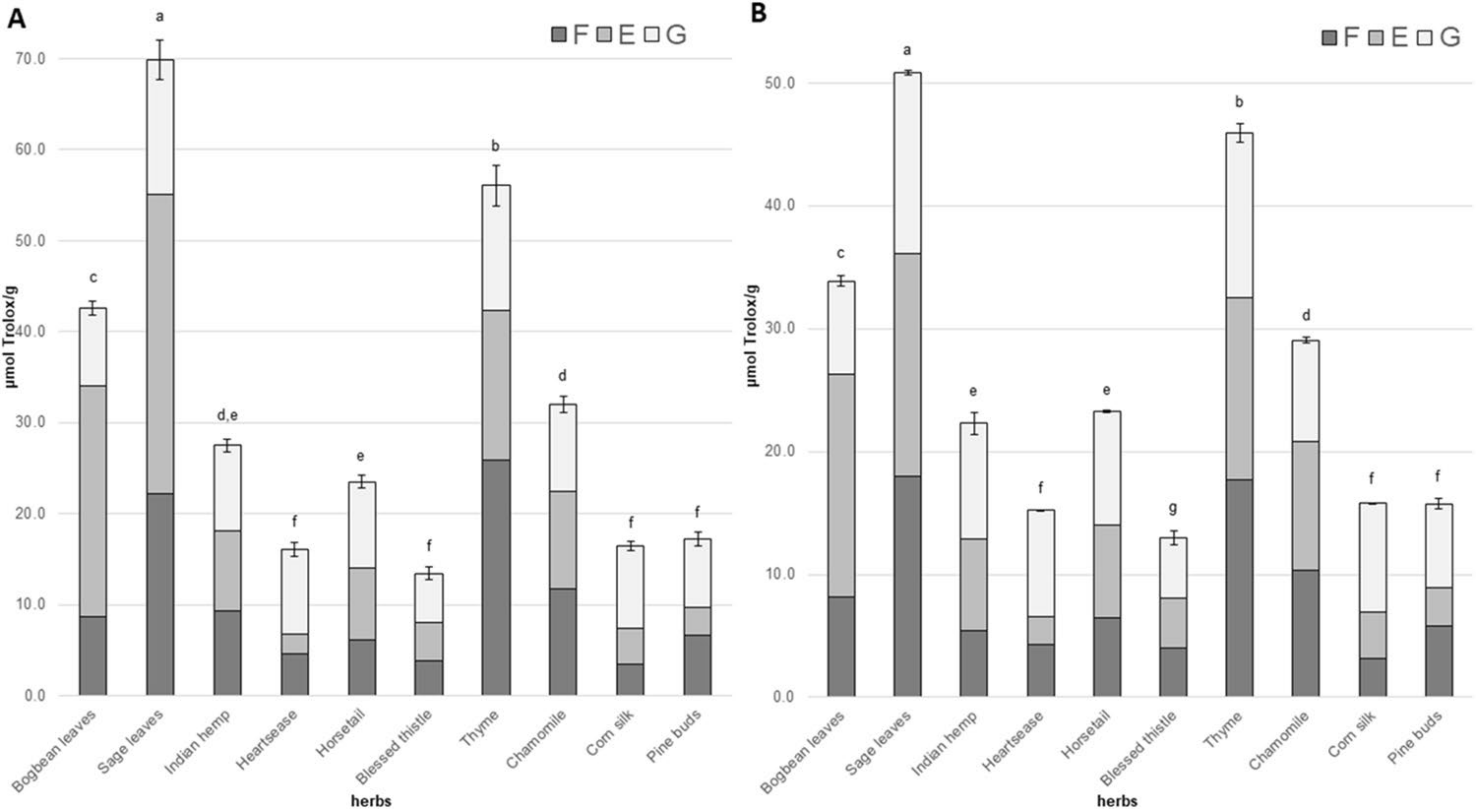
Antioxidant activity (AA) determined by ABTS (**a**) and DPPH (**b**) assays in tested herbs. F – antioxidant activity determined in non-hydrolyzed extracts; E - antioxidant activity determined in extracts after alkaline hydrolysis; G - antioxidant activity determined in extracts after acid hydrolysis

The antioxidant activity (AA) in the herb extracts determined by the ABTS method ranged from 13.46 to 69.88 *μ*mol Trolox/g. The sage leaves were characterized by the highest AA (69.88 ± 2.22 *μ*mol Trolox/g), while the lowest AA was observed in the herb of the blessed thistle (13.46 ± 0.26 *μ*mol Trolox/g). Moreover, it was observed that the AA determined by the ABTS method in the herb extracts after alkaline hydrolysis was the highest in six herbs (Indian hemp, heartsease, horsetail, blessed thistle, pine buds and corn silk; Fig. 2a). These values ranged from 33.9 (Indian hemp) to 58.14% (heartsease) of the AA determined by the ABTS test. Moreover, in the bogbean leaves and the sage leaves, these values were the highest in the extracts subjected to acid hydrolysis (59.5 and 47.0%, respectively). However, in the thyme herb (46.2%) and the chamomile (36.8%), the highest percentage contribution of AA measured by ABTS was observed in the non-hydrolyzed extracts. The high AA in the non-hydrolyzed extracts of these plants may be due to the presence of numerous volatile compounds [24]. The values of AA determined by the ABTS method were as follows: sage leaves > thyme > bogbean leaves > chamomile ≥ Indian hemp ≥ horsetail > pine buds = corn silk = heartsease = blessed thistle.

The AA of the examined extracts measured by the DPPH test ranged from 12.96 to 50.84 *μ*mol Trolox/g (Fig. 2b). The extract obtained from sage leaves (50.84 ± 0.21 *μ*mol Trolox/g) was characterized by the highest AA in the investigated herbal extracts. However, the herb of the blessed thistle was characterized by the lowest values of AA (12.96 ± 0.44 *μ*mol Trolox/g). Moreover, it was noted that the highest values of AA determined by the DPPH method were measured in herbal extracts subjected to the hydrolysis process (except thyme herb). This data may suggest that the tested herbs may show more significant biological activity *in vivo* than *in vitro*. As mentioned above, the highest values of AA in thyme were in the non-hydrolyzed extracts. The percentage contribution of AA determined in these extracts was 38.5% of the total AA of this herb. Despite this, the highest values of AA found in extracts subjected to alkaline hydrolysis were observed in the bogbean leaves (53.5%), sage leaves (35.6%) and chamomile (35.9%). However, the other tested herbs (Indian hemp, heartsease, horsetail, blessed thistle, corn silk, pine buds) were characterized by the highest value of AA determined in the extracts subjected to acid hydrolysis. Heartsease had the highest value of AA determined by the DPPH method among the extracts subjected to acid hydrolysis. In contrast, the lowest value was detected in the blessed thistle acid extracts. The investigated herbs were characterized by the following antioxidant activity determined by the DPPH method: sage leaves > thyme herb > bogbean leaves > chamomile > horsetail ≥ Indian hemp > corn silk = pine buds = heartsease > blessed thistle.

In other reports on the AA of the tested herbs, ambiguous values were observed. In a study by Mocan et al. [17], a higher AA of sage determined by ABTS and DPPH assays was determined. In contrast, Vicaș et al. [25] and Taârit et al. [16] found the lowest AA for sage determined by ABTS and DPPH tests, respectively. Moreover, other studies presented lower AA (DPPH assay) values for Indian hemp, thyme herb, chamomile and corn silk than in our study [20, 26–28]. Furthermore, lower ABTS values were observed for heartsease, thyme and corn silk [26, 29, 30]. The ambiguous findings presented in the current and previous studies may indicate that the AA of herbs depends not only on the presence of polyphenolic compounds but also on other phytochemicals with antioxidant potential.

Additionally, the correlation coefficient (r) between the AA values and the TP and TF contents was determined. The correlation coefficient between ABTS and TP was 0.941, while between ABTS and TF was 0.373. Moreover, the “r” values between the DPPH and the TP and TF were  = 0.876 and r = 0.361, respectively. The obtained values suggest that total phenolic compounds are more responsible for the antioxidant activity determined by the ABTS and DPPH assays than the flavonoid compounds themselves. The results of this study are consistent with those found in the literature [23].

### The Profile of Phenolic Compounds in Tested Herbs

In the tested material, 33 phenolic compounds were determined, of which 16 were phenolic acids, 9 were flavonoids, and eight were stilbenes (Table S1). As in the case of TP and TF contents, the available literature provides only data regarding the presence of the free form of individual phenolic compounds.

The horsetail was characterized by the richest profile of free phenolic compounds (33 compounds); while bogbean leaves were characterized by the poorest profile of these substances (16 compounds). Six compounds (naringenin, orientin, rutin, coutaric acid, caftaric acid and cinnamic acid) were present in all tested non-hydrolyzed extracts. The results in Table S1 indicate that in the five tested herbs (sage leaves, horsetail, blessed thistle, thyme and chamomile) the major compound belonged to the phenolic acids. Ferulic acid, m-hydroxybenzoic acid, chlorogenic acid, caffeic acid and syringic acid were dominant compounds in horsetail thyme, chamomile, blessed thistle and sage leaves, respectively. However, in four non-hydrolyzed herb extracts the major compounds were flavonoids. Apigenin was a major compound only in corn silk, while orientin was dominant in three herbs (bogbean leaves, Indian hemp and pine buds). Moreover, only one non-hydrolyzed sample (heartsease herb) possessed the dominant compound from the stilbenes group (E-resveratrol). Moreover, it was noted that flavonoids were the dominant group of phenols in most of the non-hydrolyzed extracts (Fig. 1S). In the non-hydrolyzed samples, the sum of phenolic compounds ranged from 0.02 ± 0.00 to 2.82 ± 0.01 mg/g (Table S2). The highest sum of phenolic compounds content in the present study was found in the chamomile flower (2.82 ± 0.01 mg/g). This value was over 1000 times higher than the total content of phenolic compounds in heartsease, blessed thistle and corn silk. The sum of free phenolic compounds in these three herbs was the lowest (0.02 ± 0.00 mg/g).

For extracts after the alkaline hydrolysis, the richest profile of phenolic compounds was found in bogbean leaves (33 compounds) (Table S3). This result is opposite to that obtained from non-hydrolyzed extracts of bogbean leaves, in which this herb was characterized by the lowest number of identified compounds (16 compounds). However, the poorest profile of phenolic compounds was found in thyme (22 compounds). Compounds from the phenolic acids group were the main substances in six herbs (sage leaves, heartsease, blessed thistle, thyme, chamomile and corn silk). Ferulic acid was the dominant compound in two herbs (chamomile and corn silk). Moreover, syringic acid was the primary phenolic acid in two herbs (sage leaves and thyme). In contrast, the primary phenolic acids in the heartsease and blessed thistle were *m*-hydroxybenzoic and caffeic acids, respectively (Table S2). In addition, the phenolic acids were the dominant group of phenols in most of the extracts after alkaline hydrolysis (Fig. 2S). However, compounds from the flavonoid group were dominant in three extracts after alkaline hydrolysis (bogbean leaves, Indian hemp and horsetail). Rutin dominated in Indian hemp, apigenin in the bogbean leaves and orientin in the horsetail. Similar to the case of the non-hydrolyzed samples, in only one herb was a compound from stilbenes dominant. E-astringin was a major compound in the pine buds, with a 21.19% contribution (Table S2). Moreover, sage leaf was characterized by the highest sum of phenolic compound released from ester bonds. The content of the compounds determined in this herb was 2.93 ± 0.00 mg/g. On the other hand, the lowest content of phenolic compounds released from ester bonds was measured in corn silk (0.01 ± 0.00 mg/g).

As shown in Table S4, the richest profile of phenolic compounds released from glycosidic bonds (acid hydrolysis) was detected in bogbean leaves (30 compounds). These data are similar to alkaline hydrolysis, which suggests that phenolic compounds in bogbean leaves are mostly present in glycosidic and ester bonds. In contrast, the lowest number of phenolic compounds was determined in sage leaves, corn silk and pine buds (24 compounds each) (Table S4). Moreover, the number of detected compounds in the sample after acid hydrolysis was higher than the number of compounds found in the non-hydrolyzed extracts(16 compounds) and in extracts after alkaline hydrolysis (22 compounds). Furthermore, the dominant compound was represented by phenolic acids in 9 herbs (Table S4), while in only one herb (corn silk) was the major compound from flavonoids (luteolin). As in the extracts after alkaline hydrolysis, phenolic acids were the dominant group of phenols in extracts after acid hydrolysis (Fig. 3S). The sum of phenolic compounds released from glycosidic bonds ranged from 0.02 ± 0.00 to 0.91 ± 0.00 mg/g. The highest content of these compounds were found in sage leaf (0.91 ± 0.00 mg/g), while the lowest is thyme (0.02 ± 0.00 mg/g).

Most studies dedicated to the phenolic compound profiles refer to their free form [18, 25, 31, 32]. Roby et al. [31] analyzed chamomile phenolic compounds such as neochlorogenic acid, chlorogenic acid, gallic acid, caffeic acid, *p*-coumaric acid, ferulic acid, ferulic acid, 1,5-dicaffeoylquinic acid, hesperidin, cinnamic acid, acid rosemary, quercetin and apigenin. The dominant compound in the cited study was quercetin, whose share was 11.56%, while the chlorogenic acid contribution in the cited study was 3.24%. In contrast, the current study showed that the percentage contribution of quercetin in the chamomile was at a lower level (0.09%), which could have been caused by different growing conditions, species, or the extraction method used [31]. In the case of sage, the phenolic compound profile consisted of phenolic compounds, *i.e*., gallic acid, chlorogenic acid, caffeic acid, quinic acid, *p*-coumaric acid, quercetin, ferulic acid, carnosic acid, cinnamic acid, rosmarinic acid, apigenin, naringin, and luteolin [31]. A study by Roby et al. [31] showed that the dominant compound were ferulic acid (18.79%) and rosmarinic acid (17.85%). However, in the current study, rosmarinic acid was not identified, and the content of ferulic acid was approximately four times lower than in the cited studies. In addition, Roby et al. [31] also investigated the profile of phenolic compounds in thyme. Those authors found that the dominant phenolic compound was cinnamic acid, with a percentage contribution of 28.54%. For comparison, in our study, *m*-hydroxybenzoic acid and *p*-hydroxybenzoic acid (15.04 and 15.02%, respectively) were compounds determined in thyme (Table S2). A small number of phenolic compounds (rutin, ferulic acid, *p*-coumaric acid, epicatechin, caffeic acid, syringic acid, vanillic acid and protocatechuic acid) were also found in horsetail by Čanadanović-Brunet et al. [33]. Moreover, the results of a study by Oliva et al. [34] pointed to the presence of 34 phenolic compounds in Indian hemp. In comparison, 15 out of 34 phenolic compounds detected by Oliva et al. [34] were also identified in the current study. As mentioned above, compounds from stilbenes dominated only in non-hydrolyzed extracts of heartsease. A study conducted by Sadeghnia et al. [35] showed that kaempferol, luteolin, Z-resveratrol and E-resveratrol were present in heartsease. In turn, Paun et al. [19] showed the presence of 14 phenolic compounds in blessed thistle (isoquercetin, quercetin, rutin, kaempferol, luteolin, apigenin, caffeic acid, rosmarinic acid, ferulic acid, chlorogenic acid, coumaric acid, *p*-coumaric acid, daidzein and genistein). Moreover, the dominant compound in the studies conducted by Paun et al. [19] was chlorogenic acid. Furthermore, a small number of phenolic compounds (caffeic acid, gallic acid and Z-resveratrol and E-resveratrol) were also detected in corn silk [20], whose presence was also confirmed in this study. As in the case of two other herbs (bogbean leaves and Indian hemp), orientin had the highest percentage (38.05 and 25.50%, respectively). The four compounds present in this herb (chlorogenic acid, coumaric acid, 3,4-dihydrophenylacetic acid and syringic acid) were not detected. Moreover, pine buds and horsetail herb both contained all the stilbenes. The different number of phenolic compounds identified in herbs may result from varietal diversity, the influence of vegetation season, climatic and cultivation conditions, and extraction and analytical methods [3]. However, the results obtained in the current study indicate that the tested herbs may be a valuable source of phenolic compounds with their own unique profiles.

## Conclusions

This is the first study to present the composition of free and conjugated phenolic compounds in 10 different herbs. The study showed that each tested herb possesses its own fingerprint of phenolic compounds. The TP, TF and total content of individual compounds varied significantly among herbs. Moreover, each hydrolysed and non-hydrolyzed herb extract was characterized by a specific level of phenolic compounds. The study also showed that the bioactive compounds in herbs are primarily present in bound forms rather than in free forms. In addition, each herb was characterized by a specific and unique antioxidant activity. The results of the study indicated that the tested herbs are a valuable source of phenolic acids, flavonoids and stilbenes with high antioxidant activity. Moreover, the phenolic compound profile characteristics and antioxidant activity of different herbs may encourage the wider use of these products in the food industry and the development of new functional foods.

## Supplementary Information

ESM 1 (DOCX 1009 kb)

## Data Availability

All data generated or analyzed for this study are included in this published article.

## Abbreviations

AA: Antioxidant activity
ABTS: Diammonium salt
DPPH: 2,2-diphenyl-1-picrylhydrazyl
GAE: gallic acid equivalents
TP: Total phenolic content
TF: Total flavonoid content
QE: Quercetin equivalents
