# Supplementary material for: Antioxidant Activity and Profile of Phenolic Compounds in Selected Herbal Plants
Source: Plant Foods Hum Nutr. 2022 Jul 2;77(3):383–9. doi: 10.1007/s11130-022-00989-w (PMC9463321; doi:10.1007/s11130-022-00989-w)
Supplement: Supplementary file 1 — (DOCX 1009 kb) [file 11130_2022_989_MOESM1_ESM.docx]

**Supplementary Information**

Kamil Foss, Katarzyna E. Przybyłowicz, Tomasz Sawicki^*^

Department of Human Nutrition, Faculty of Food Sciences, University of Warmia and Mazury in Olsztyn, Słoneczna 45F, 10-719 Olsztyn, Poland

* Corresponding author: Tomasz Sawicki, [tomasz.sawicki@uwm.edu.pl](mailto:tomasz.sawicki@uwm.edu.pl)

**Materials and methods**

*Chemicals and reagents*

The following chemicals and reagents were purchased from Sigma Chemical Co. (St. Louis, MO, USA): Folin phenol reagent, 6-hydroxyl-2,5,7,8-tetramethyl-chroman-2-carboxylic acid (Trolox), 2,2′-azinobis(3-ethylbenzothiazoline-6-sulphonic acid) diammonium salt (ABTS), 2,2-diphenyl-1-picrylhydrazyl (DPPH), AlCl_3_, NaNO_2_, , and HPLC-grade solvents (water, acetonitrile, methanol and formic acid). Standards of phenolic acids (gallic, caffeic, chlorogenic, ferulic, *p*-coumaric, sinapic, *t*-cinnamic, caftaric, *m*-hydrocxybenzoic, protocatechuic, coutaric, syringic and vanillic acids), flavonoids (quercetin, kaempferol, naringenin, rutin, vitexin, apigenin, luteolin, epicatechin and orientin) and stilbenes (resveratrol) were purchased from Sigma Chemical Co. (St. Louis, MO, USA).

*Plant material and sample preparation*

The analyzed samples included ten herbs that were available in a specialized herbal store in Olsztyn, Poland market in 2021. The herbs obtained (100 g for each sample) were: bogbean leaves (*Menyanthidis trifoliatae* L.), sage leaves (*Salviae folium*), whole Indian hemp (*Cannabis sativa* L.), whole heartsease (*Violae tricoloris*), whole horsetail (*Equisetum arvense* L.), whole blessed thistle (*Cnici benedicti*), whole thyme (T*hymus serpyllum* L.), chemomile flower (*Matricariae recutita* L.), corn silk (*Stigma maydis*), pine buds (*Pinus silvestris*). The samples obtained were pulverized and stored at −24 °C until the analysis. All investigated samples belonged to the eight different plant families: *Menyanthaceae* (bogbean leaves), *Lamiaceae* (sage leaves and thyme), *Apocynaceae* (Indian hemp), *Violaceae* (heartsease), *Equisetaceae* (horsetail), *Asteraceae* (blessed thistle and chemomile), *Poaceae* or *Gramineae* (corn silk) and *Pinaceae* (pine buds).

*Extraction of free and conjugated polyphenolic compounds*

Phenolics were extracted according to the method described by Płatosz et al. [1]. Herb samples (0.1 g) were extracted with 2 mL of water/methanol/formic acid mixture (volume ratio: 19.9/80/0.1) by stirring overnight at room temperature (Thermal Shake Touch; VWR, Radnor, PA, USA). Afterwards the obtained solutions were centrifuged for 20 minutes (14,000 × g, at 4°C, Centrifuge Mega Star 600R, VWR). The resulting supernatants were stored at -80 °C until future analysis.

Phenolics (free and those conjugated) were isolated from the obtained extracts according to the following procedure. In the first step, the extracts of herbs were transferred to 15-mL glass tubes and evaporated to dryness under a nitrogen atmosphere. The sediments samples were dissolved in 400 µL of distilled water and adjusted to pH 2.0 with 6 M HCl. The free forms of the analyzed compounds were extracted three times with 2mL of diethyl ether by vortexing (30 s) and sonification (30 s). Next, the samples were centrifuged for 5 min (5,000 x g at 4 °C), and the ether extract was collected and evaporated to dryness under nitrogen. In the subsequent step, a volume of 4M NaOH was added to the remaining extract, and the obtained mixture was placed under a nitrogen atmosphere and hydrolyzed for 4 h at room temperature. After acidification to pH 2.0 using 6 M HCl, the compounds freed from soluble esters were extracted with 2 mL of diethyl ether following the procedure described above. In the third step, a volume of 6M HCl was added to the remaining extract, and the mixture was hydrolyzed for 1 h at 100 °C. The pH of the solution containing compounds liberated from soluble glycosides was adjusted using 8 M NaOH, and then the compounds released from soluble glycosides were extracted in diethyl ether. After centrifugation the ether extract was collected. Each ether extract was evaporated to dryness under nitrogen and stored at –20 °C until the future analysis. Before analysis, the dry residues containing free and conjugated phenolic compounds were reconstituted in 100 µL of 80% (v/v) methanol containing 0.1% (v/v) formic acid and centrifuged for 20 minutes (14,000 × g at 4°C).

*Determination of total phenolic (TP) and total flavonoid (TF) contents*

The total phenolic (TP) content was carried out using the Folin-Ciocalteu reagent according to the procedure described by Sawicki et al. [2]. A mixture containing 15 µL of appropriately diluted extract fractions and 240 µL of Folin’s phenol reagent were placed into wells of microplates and incubated for 10 min at room temperature (RT). Next, 15 µL of 20% sodium carbonate was added and shaken. Absorbance was measured at 765 nm using a microplates reader (FLUOstar Omega, BMG LABTECH, Ortenberg, Germany). Obtained result were expressed as mg gallic acid equivalents (GAE) per gram of sample.

The assay of total flavonoid (TF) content was carried out according to the procedure described by Sawicki et al. [2]. A mixture containing 25 µL of extract fractions and 75 µL of ethanol (v/v) was mixed with 5 µL of 10% AlCl_3_*6H_2_O as well as 5 µL of 1 mol/L potassium acetate. Next 140 µL of deionized water was added to each well, mixed and left for 30 min at RT. After this time, the absorbance was measured at 415 nm using a microplate reader (FLUOstar Omega, BMG LABTECH). The TFC of each samples was calculated by interpolating with the calibration curve built with quercetin as a standard. Results are expressed as mg quercetin equivalents (QE) per gram of sample. All measurements were performed in triplicates.

*Chromatographic analysis*

Determination of polyphenolic compounds in each fraction of herb extracts were carried out using the liquid chromatograph (LC) coupled with the Ultra-High Resolution Qq-Time-Of-Flight type of mass spectrometer (MS; Impact II, Bruker, Billerica, MA, USA) according to the method described by Tomczyk et al. [3] with some modifications.. The analysis was based on scanning in negative ionization mode in high sensitivity mode. The separation of the compounds was carried out on Kinetex C18 100 x 1 mm column (Phenomenex, Torrance, CA, USA) at 40 °C with the flow rate of 0.35 mL/min. The elution was conducted using a solvent gradient system consisting of solvent A (0.1% formic acid aqueous solution) and solvent B (0.1% formic acid in 40% acetonitrile). The system was run with the following gradient program: 5% B (0-0.5 min), 5-95% B (0.5-6.5 min), 95% B (6.5-8 min), 95-5% B (8-10 min) and 5% B (10-13 min). The injection volume of the samples was 10 µL. Optimal identification of phenolic compounds was achieved under the following parameters: capillary voltage of 3000 V, dry temperature 350°C, and dry gas flow rate of 8 L/min, nebulizing gas 40 psi. The MS functioned in mass rage from 50 to 1300 m/z. The collision-induced dissociation (CID), ion energy (IE) and collision energy (CE) for the MS experiment were 5 eV, 8 eV and 20 eV, respectively. Characterization of the individual polyphenolic compounds was performed on the basis of the retention time, fragment ions, and comparison of data obtained with literature findings [2, 4-6]. The MS data of herbs polyphenols are presented in Table S1

The quantity of phenolic compounds was calculated from HPLC-TOF-MS/MS peak area against the commercially available standards. Calibration solutions were prepared from the phenolics stock standard solutions in 1 ml flasks by replenishing the flasks with 80% methanol solution. The phenolic compound concentrations of the solutions were from 0.01 to 100 μg/mL. The method of least squares was used to obtain the equations of calibration curves (y = ax + b). A goodness of fit being given by the coefficient of determination (R^2^), which is the evidence of linearity for all analyzed phenolic compounds in the concentration range from 0.01 to 100 μg/mL (Table 1). The limit of detection value (LOD) was calculated based on the standard deviations of the series of the phenolic compounds' standard solutions. A linear relationship was determined, linking the computed values of standard deviations with concentrations. LOD values were determined according to equation 1, while the limit of quantification (LOQ) was calculated as a triplicate of LOD value (Table 1).

LOD = 3 * s (1)

where:

s - the intercept of the linear correlation between the standard deviations of phenolic standards and their concentrations [7].

*Determination of antioxidant activity*

The ABTS and DPPH assays described by Sawicki et al. [2] were used to evaluate the antioxidant activity of the obtained extracts. The absorbance was measured at 734 nm (ABTS assay) and 517 nm (DPPH assay) using a microplate reader (FLUOstar Omega, BMG LABTECH). Results were presented as µmol Trolox per gram of sample. All measurements were performed in triplicates.

*Statistical analysis*

The data are presented as mean values ± standard deviations of triplicate measurement. The differences between samples were analyzed by a one-way ANOVA with Tukey’s test (p < 0.05). The statistical analysis was performed using STATISTICA 13.0 (StatSoft Inc., Tulsa, OK, USA).

**References**

1. Płatosz N, Sawicki T, Wiczkowski W (2020) Profile of phneolic acids and flavonoids of red beet and its fermentation products. Dose long-term consumption of fermented beetroots juice affect phenolics profile in human blood plasma and urine? Polish J Food Nutr Sci 70:55-65. <https://doi.org/10.31883/pjfns/116613>
2. Sawicki T, Starowicz M, Kłębukowska L, Hanus P (2022) The profile of polyphenolic compounds, contents of total phenolics and flavonoids, and antioxidant and antimicrobial properties of bee products. Molecules 27:1301. https://doi.org/10.3390/molecules27041301
3. Tomczyk M, Miłek M, Sidor E, Kapusta I, Litwińczuk W, Puchalski C, Dżugan M (2020) The effect of adding the leaves and fruits of Morus alba to rape honey on its antioxidant properties, polyphenolic profile, and amylase activity. Molecules 25:84. https://doi.org/10.3390/molecules25010084
4. Gabaston J, Valls Fonayet, J Franc, et al (2020) Characterization of stilbene composition in grape berries from wild vitis species in year-to-year harvest. J Agric Food Chem 68:13408-13417. [https://doi.org/10.1021/acs.jafc.0c04907](https://doi.org/10.1021/acs.jafc.0c04907)
5. Tarapatskyy M, Gumienna A, Sowa P, Kapusta I, Puchalski C (2021) Bioactive phenolic compounds from *primula veris* L.: Influence of the extraction conditions and purification. Molecules, 26:997. <https://doi.org/10.3390/molecules26040997>
6. Singh A, Bajpai V, Kumar S, Sharma KR, Kumar B (2016) Profiling of gallic and ellagic acid derivatives in different plant parts of T*erminalia arjuna* by HPLC-ESI-QTOF-MS/MS. Nat Prod Commun 11:239-244. <https://doi.org/10.1177/1934578X1601100227>
7. Konieczka P, Namieśnik J, Zygmunt B, Bulska E, Świtaj-Zawadka A, Naganowska A, Kremer E, Rompa M. (2004) Ocena i kontrola jakości wyników analitycznych (Chapt. 8.7). CEEAM, Gdańsk, Poland.

|  | **No** | **Compounds** | **R_t_**  **[min]** | **[M]^-^**  **(m/z)** | **MS/MS**  **(m/z)** | **a** | **R^2^** | **LOD**  **[μg/g]** | **LOQ**  **[ μg/g]** |
| --- | --- | --- | --- | --- | --- | --- | --- | --- | --- |
| **Phenolic acids** | 1 | *p*-coumaric acid | 2.60 | 163 | 119/93 | 3E-8 | 0.998 | 0.070 | 0.210 |
|  | 2 | *m*-hydroxybenzoic acid | 2.70 | 137 | 93/65 | 1E-8 | 0.999 | 0.011 | 0.033 |
|  | 3 | coumaric acid | 2.71 | 163 | 119/93 | 2E-8 | 0.997 | 0.061 | 0.183 |
|  | 4 | *t*-cinnamic acid | 2.73 | 147 | 119/109/103 | 2E-7 | 0.999 | 0.019 | 0.058 |
|  | 5 | caftaric acid | 2.74 | 311 | 179 | 2E-7 | 0.999 | 0.061 | 0.183 |
|  | 6 | coutaric acid | 4.60 | 295 | 163 | 3E-7 | 0.998 | 0.031 | 0.093 |
|  | 7 | gallic acid | 5.30 | 169 | 125 | 2E-7 | 0.998 | 0.016 | 0.048 |
|  | 8 | sinapic acid | 6.20 | 223 | 208/179/164 | 2E-7 | 0.998 | 0.078 | 0.235 |
|  | 9 | 3.4-didydrohyphenylacetic acid* | 6.40 | 169 | 125/109/95 | 1E-7 | 0.999 | 0.015 | 0.046 |
|  | 10 | protocatchuic acid | 6.83 | 153 | 91/81 | 4E-8 | 0.999 | 0.101 | 0.303 |
|  | 11 | chlorogenic acid | 7.80 | 353 | 191/179 | 9E-8 | 0.999 | 0.020 | 0.061 |
|  | 12 | *p*-hydroxybenzoic acid | 7.82 | 137 | 98/93 | 1E-8 | 0.999 | 0.011 | 0.033 |
|  | 13 | caffeic acid | 8.50 | 179 | 135/107 | 1E-7 | 0.999 | 0.035 | 0.104 |
|  | 14 | syringic acid | 10.02 | 197 | 182/153 | 2E-8 | 0.997 | 0.045 | 0.136 |
|  | 15 | ferulic acid | 10.21 | 193 | 178/134 | 3E-8 | 0.998 | 0.018 | 0.054 |
|  | 16 | vanillic acid | 11.00 | 167 | 152 | 2E-7 | 0.999 | 0.017 | 0.052 |
| **Flavonoids** | 17 | epicatechin | 7.11 | 289 | 245/203/109 | 6E-8 | 0.999 | 0.032 | 0.096 |
|  | 18 | orientin | 8.82 | 447 | 357/339/296 | 1E-6 | 0.997 | 0.144 | 0.432 |
|  | 19 | vitexin | 9.30 | 431 | 323/311/283 | 2E-8 | 0.999 | 0.051 | 0.153 |
|  | 20 | apigenin | 9.41 | 269 | 225/151/117 | 1E-7 | 0.998 | 0.015 | 0.045 |
|  | 21 | naringenin | 9.43 | 271 | 177/151/119/107/93 | 3E-8 | 0.998 | 0.016 | 0.048 |
|  | 22 | luteolin | 9.70 | 285 | 151/133 | 7E-8 | 0.998 | 0.064 | 0.192 |
|  | 23 | rutin | 9.91 | 609 | 463/301 | 7E-8 | 0.999 | 0.011 | 0.033 |
|  | 24 | quercetin | 10.30 | 301 | 179/151 | 3E-8 | 0.999 | 0.025 | 0.075 |
|  | 25 | kaempferol | 11.11 | 285 | 185/93 | 5E-8 | 0.999 | 0.024 | 0.072 |
| **Stilbenes*** | 26 | E-astringin | 2.72 | 405 | 243/201 |  |  |  |  |
|  | 27 | E-isorhapontin | 5.11 | 419 | 257/241 |  |  |  |  |
|  | 28 | E-resveratrol | 6.43 | 227 | 143/185 | 4E-7 | 0.998 | 0.031 | 0.093 |
|  | 29 | E-piceid | 7.70 | 389 | 227/143 |  |  |  |  |
|  | 30 | Z-piceid | 9.30 | 389 | 227/143 |  |  |  |  |
|  | 31 | E-resveratrol | 9.82 | 227 | 143/185 | 4E-7 | 0.998 | 0.031 | 0.093 |
|  | 32 | Z-astringin | 10.41 | 405 | 243/201 |  |  |  |  |
|  | 33 | ampelopsin A | 11.04 | 469 | 451 |  |  |  |  |

Table S1. The MS data, parameters of calibration curve, LOD and LOQ of phenolic compounds identified in the tested herbs.

Abbreviations: R_t_ – retention time; [M]^-^ (m/z) – parent ion; MS/MS (m/z) – fragment ion; a – calibration slope; R^2^ – coefficient of determination; LOD – limit of detection; LOQ – limit of quantification. * The values of the resveratrol calibration curve were used to calculate the content of individual stilbenes.

Table S2. Content and composition of free phenolic compounds in tested herbs.

|  | Compounds | % of contribution in sum of phenolic compounds | | | | | | | | | |
| --- | --- | --- | --- | --- | --- | --- | --- | --- | --- | --- | --- |
|  |  | Herbs | | | | | | | | | |
|  |  | Bogbean leaves | Sage leaves | Indian hemp | Heartsease | Horsetail | Blessed thistle | Thyme | Chamomile | Corn silk | Pine buds |
| **Phenolic acids** | protocatchuic acid | 0.00 | 0.20 | 0.50 | 1.50 | 0.66 | 9.88 | 3.95 | 0.37 | 2.99 | 6.01 |
|  | ferulic acid | 1.72 | 4.61 | 1.99 | 0.00 | 21.78 | 11.79 | 1.05 | 15.47 | 4.08 | 2.00 |
|  | *m*-hydroxybenzoic acid | 0.66 | 0.00 | 0.00 | 0.53 | 1.05 | 7.39 | 15.04 | 0.00 | 3.18 | 1.08 |
|  | chlorogenic acid | 1.15 | 0.02 | 1.49 | 0.22 | 0.02 | 1.02 | 13.15 | 45.32 | 0.40 | 0.00 |
|  | *p*-coumaric acid | 0.23 | 0.32 | 1.12 | 0.41 | 9.01 | 0.00 | 0.52 | 0.05 | 0.30 | 0.89 |
|  | coumaric acid | 0.60 | 0.18 | 0.45 | 0.39 | 1.84 | 0.00 | 0.33 | 0.03 | 0.88 | 0.00 |
|  | *t*-cinnamic acid | 0.43 | 0.11 | 0.74 | 1.89 | 0.31 | 1.06 | 1.32 | 0.06 | 2.95 | 0.12 |
|  | caffeic acid | 0.00 | 2.97 | 6.84 | 0.23 | 5.23 | 18.11 | 11.19 | 0.69 | 0.77 | 1.48 |
|  | syringic acid | 0.00 | 74.00 | 0.00 | 0.00 | 3.38 | 1.87 | 4.52 | 0.00 | 6.21 | 0.00 |
|  | galic acid | 0.00 | 0.03 | 0.00 | 0.25 | 0.23 | 0.26 | 0.00 | 0.07 | 0.14 | 0.15 |
|  | sinapic acid | 0.00 | 0.03 | 0.73 | 0.10 | 0.07 | 0.00 | 0.40 | 0.10 | 0.00 | 0.08 |
|  | *p*-hydroxybenzoic acid | 0.00 | 1.99 | 1.75 | 4.41 | 1.35 | 7.40 | 15.02 | 0.43 | 3.14 | 1.07 |
|  | caftaric acid | 0.42 | 0.33 | 3.33 | 3.57 | 0.47 | 3.39 | 1.87 | 0.33 | 5.74 | 0.99 |
|  | 3.4-didydrohyphenolacetic acid | 0.00 | 0.03 | 0.00 | 0.11 | 0.23 | 0.26 | 0.00 | 0.00 | 0.00 | 0.00 |
|  | coutaric acid | 0.14 | 0.20 | 0.73 | 10.07 | 1.08 | 13.38 | 0.80 | 0.28 | 1.44 | 0.03 |
|  | vanillic acid | 0.00 | 0.22 | 1.08 | 1.05 | 0.55 | 1.37 | 1.47 | 0.27 | 16.37 | 5.98 |
| **Flavonoids** | luteolin | 0.00 | 1.25 | 0.14 | 0.80 | 1.07 | 1.18 | 3.01 | 0.72 | 3.93 | 15.18 |
|  | epicatechin | 0.00 | 0.00 | 1.66 | 0.22 | 4.56 | 0.11 | 2.92 | 0.20 | 1.12 | 1.92 |
|  | vitexin | 0.00 | 0.19 | 11.72 | 0.35 | 7.07 | 0.35 | 1.97 | 26.99 | 0.00 | 0.43 |
|  | rutin | 22.66 | 0.88 | 17.45 | 12.78 | 5.68 | 0.17 | 0.91 | 0.02 | 0.57 | 0.09 |
|  | quercetin | 13.52 | 0.06 | 0.00 | 2.29 | 18.01 | 3.97 | 2.80 | 0.09 | 4.86 | 1.61 |
|  | apigenin | 0.70 | 0.00 | 1.23 | 9.61 | 3.42 | 1.45 | 0.46 | 1.54 | 29.41 | 0.82 |
|  | kaempferol | 8.63 | 1.24 | 0.79 | 0.00 | 0.28 | 6.83 | 3.12 | 0.73 | 3.88 | 15.25 |
|  | orientin | 38.05 | 10.96 | 25.53 | 3.44 | 6.18 | 0.66 | 4.82 | 4.43 | 2.21 | 42.95 |
|  | naringenin | 4.32 | 0.11 | 2.97 | 0.98 | 2.78 | 0.36 | 1.41 | 1.34 | 4.27 | 1.13 |
| **Stilbenes** | E-piceid | 5.55 | 0.00 | 0.70 | 0.13 | 0.03 | 0.16 | 0.00 | 0.02 | 0.00 | 0.02 |
|  | Z-piceid | 0.00 | 0.00 | 14.05 | 0.11 | 2.43 | 0.36 | 0.00 | 0.11 | 0.00 | 0.10 |
|  | E-resveratrol | 1.25 | 0.06 | 1.93 | 41.91 | 0.11 | 5.68 | 0.86 | 0.14 | 0.00 | 0.27 |
|  | E-resveratrol | 0.00 | 0.01 | 0.13 | 1.62 | 0.17 | 0.41 | 3.62 | 0.04 | 0.00 | 0.03 |
|  | E-astringin | 0.00 | 0.00 | 0.15 | 0.34 | 0.31 | 0.00 | 2.77 | 0.00 | 0.00 | 0.16 |
|  | Z-astringin | 0.00 | 0.00 | 0.58 | 0.30 | 0.22 | 0.00 | 0.13 | 0.00 | 0.35 | 0.06 |
|  | E-isohapontin | 0.00 | 0.02 | 0.20 | 0.40 | 0.17 | 0.88 | 0.56 | 0.04 | 0.12 | 0.02 |
|  | ampelopepsin A | 0.00 | 0.00 | 0.00 | 0.00 | 0.24 | 0.25 | 0.00 | 0.12 | 0.69 | 0.09 |
| Sum of phenolic compounds [mg/g]^*^ | | 0.79 ± 0.00^b^ | 0.51 ± 0.01^b,c^ | 0.06 ± 0.00^c^ | 0.02 ± 0.00^c^ | 0.43 ± 0.00^b,c^ | 0.02 ± 0.00^c^ | 0.44 ± 0.01^b,c^ | 2.82 ± 0.01^a^ | 0.02 ± 0.00^c^ | 0.24 ± 0.00^b,c^ |

* Data are expressed as mean ± SD (n = 3). Means in line related to the sum of phenolic compounds for each herb followed by the different letters are significantly different (P < 0.05).

Table S3. Content and composition of free phenolic compounds released from glycosidic bonds.

|  | Compounds | % of contribution in sum of phenolic compounds | | | | | | | | | |
| --- | --- | --- | --- | --- | --- | --- | --- | --- | --- | --- | --- |
|  |  | Herbs | | | | | | | | | |
|  |  | Bogbean leaves | Sage leaves | Indian hemp | Heartsease | Horsetail | Blessed thistle | Thyme | Chamomile | Corn silk | Pine buds |
| **Phenolic acids** | protocatchuic acid | 0.73 | 0.00 | 7.57 | 1.94 | 0.84 | 1.66 | 1.63 | 10.07 | 0.00 | 1.15 |
|  | ferulic acid | 1.90 | 7.62 | 2.40 | 1.76 | 2.60 | 1.11 | 1.51 | 45.57 | 19.38 | 1.14 |
|  | *m*-hydroxybenzoic acid | 1.50 | 1.37 | 2.42 | 29.64 | 0.00 | 2.86 | 0.71 | 1.70 | 1.04 | 0.96 |
|  | chlorogenic acid | 0.55 | 0.04 | 0.00 | 0.00 | 0.06 | 1.16 | 2.97 | 0.00 | 0.96 | 0.60 |
|  | *p*-coumaric acid | 1.47 | 0.55 | 4.94 | 1.37 | 12.67 | 20.04 | 1.01 | 0.14 | 10.64 | 5.61 |
|  | coumaric acid | 1.20 | 0.00 | 5.14 | 0.58 | 8.64 | 10.36 | 0.00 | 7.45 | 0.93 | 2.78 |
|  | *t*-cinnamic acid | 2.55 | 0.09 | 0.23 | 0.66 | 0.42 | 0.66 | 0.13 | 0.25 | 3.58 | 0.00 |
|  | caffeic acid | 30.64 | 26.52 | 12.52 | 1.60 | 20.85 | 23.93 | 26.57 | 17.01 | 6.53 | 0.33 |
|  | syringic acid | 1.27 | 29.51 | 0.10 | 0.11 | 0.00 | 0.00 | 26.80 | 0.47 | 1.86 | 0.00 |
|  | galic acid | 0.37 | 0.00 | 1.18 | 0.07 | 0.24 | 0.27 | 0.00 | 0.77 | 1.44 | 0.00 |
|  | sinapic acid | 0.18 | 0.15 | 0.31 | 1.64 | 0.32 | 0.35 | 0.00 | 0.01 | 2.54 | 0.24 |
|  | *p*-hydroxybenzoic acid | 4.64 | 1.38 | 2.34 | 23.43 | 0.62 | 2.92 | 5.87 | 1.75 | 0.74 | 0.95 |
|  | caftaric acid | 4.69 | 0.38 | 0.91 | 2.74 | 1.32 | 3.89 | 1.54 | 0.56 | 10.34 | 1.35 |
|  | 3.4-didydrohyphenolacetic acid | 0.37 | 0.32 | 0.00 | 0.00 | 0.24 | 0.00 | 0.00 | 0.00 | 1.10 | 0.00 |
|  | coutaric acid | 0.19 | 0.90 | 1.28 | 7.21 | 0.19 | 2.96 | 0.65 | 0.50 | 0.92 | 1.49 |
|  | vanillic acid | 1.41 | 0.00 | 0.19 | 0.00 | 0.19 | 0.12 | 0.88 | 1.21 | 5.14 | 0.00 |
| **Flavonoids** | luteolin | 0.33 | 1.84 | 0.98 | 4.15 | 0.50 | 2.39 | 0.73 | 0.87 | 0.65 | 0.85 |
|  | epicatechin | 0.39 | 0.12 | 2.07 | 0.42 | 0.99 | 1.42 | 0.00 | 0.00 | 9.73 | 3.00 |
|  | vitexin | 0.20 | 6.75 | 2.83 | 0.19 | 1.66 | 0.12 | 0.83 | 0.05 | 0.96 | 0.00 |
|  | rutin | 0.74 | 0.17 | 16.72 | 2.65 | 0.27 | 0.71 | 0.00 | 0.00 | 0.29 | 0.24 |
|  | quercetin | 3.26 | 0.34 | 0.16 | 7.17 | 14.37 | 1.60 | 0.00 | 0.50 | 0.89 | 0.25 |
|  | apigenin | 30.69 | 1.56 | 12.94 | 0.97 | 4.76 | 6.24 | 23.51 | 1.25 | 11.67 | 19.49 |
|  | kaempferol | 0.33 | 1.79 | 1.11 | 2.93 | 0.11 | 0.35 | 0.73 | 0.90 | 0.53 | 1.06 |
|  | orientin | 7.26 | 16.28 | 6.78 | 0.48 | 25.61 | 1.76 | 1.41 | 7.09 | 5.03 | 9.67 |
|  | naringenin | 1.07 | 0.67 | 12.32 | 2.86 | 1.64 | 10.26 | 0.65 | 1.01 | 1.44 | 12.09 |
| **Stilbenes** | E-piceid | 0.58 | 0.37 | 0.00 | 0.62 | 0.00 | 0.87 | 0.00 | 0.03 | 0.78 | 0.02 |
|  | Z-piceid | 0.80 | 0.19 | 0.00 | 2.51 | 0.00 | 0.06 | 0.00 | 0.02 | 0.86 | 0.00 |
|  | E-resveratrol | 0.10 | 0.00 | 0.02 | 0.67 | 0.16 | 0.23 | 0.00 | 0.26 | 0.00 | 0.10 |
|  | E-resveratrol | 0.12 | 0.00 | 0.05 | 0.97 | 0.24 | 0.29 | 0.54 | 0.04 | 0.00 | 0.04 |
|  | E-astringin | 0.18 | 0.10 | 0.42 | 0.00 | 0.38 | 1.12 | 0.00 | 0.00 | 0.00 | 21.19 |
|  | Z-astringin | 0.03 | 0.03 | 1.58 | 0.00 | 0.09 | 0.15 | 0.11 | 0.00 | 0.00 | 15.17 |
|  | E-isohapontin | 0.15 | 0.81 | 0.07 | 0.58 | 0.02 | 0.17 | 1.01 | 0.02 | 0.00 | 0.21 |
|  | ampelopepsin A | 0.10 | 0.14 | 0.42 | 0.09 | 0.00 | 0.00 | 0.23 | 0.49 | 0.00 | 0.00 |
| Sum of phenolic compounds [mg/g]^*^ | | 1.56 ± 0.01^b^ | 2.93 ± 0.00^a^ | 0.22 ± 0.01^d^ | 0.04 ± 0.00^f^ | 0.09 ± 0.00^e^ | 0.04 ± 0.00^f^ | 0.77 ± 0.00^c^ | 1.56 ± 0.00^b^ | 0.01 ± 0.00^g^ | 0.08 ± 0.00^e^ |

* Data are expressed as mean ± SD (n = 3). Means in line related to the sum of phenolic compounds for each herb followed by the different letters are significantly different (P < 0.05).

Table S4. Content and composition of free phenolic compounds released from esters bonds.

|  | Compounds | % of contribution in sum of phenolic compounds | | | | | | | | | |
| --- | --- | --- | --- | --- | --- | --- | --- | --- | --- | --- | --- |
|  |  | Herbs | | | | | | | | | |
|  |  | Bogbean leaves | Sage leaves | Indian hemp | Heartsease | Horsetail | Blessed thistle | Thyme | Chamomile | Corn silk | Pine buds |
| **Phenolic acids** | protocatchuic acid | 23.37 | 1.83 | 1.86 | 56.74 | 4.44 | 7.25 | 12.62 | 25.25 | 2.83 | 0.18 |
|  | ferulic acid | 0.67 | 4.58 | 33.19 | 1.88 | 0.06 | 2.43 | 0.84 | 3.74 | 22.28 | 0.52 |
|  | *m*-hydroxybenzoic acid | 0.18 | 0.00 | 0.24 | 0.00 | 38.43 | 5.90 | 16.07 | 0.13 | 0.00 | 1.37 |
|  | chlorogenic acid | 0.12 | 2.37 | 0.10 | 0.00 | 0.11 | 0.46 | 0.50 | 0.00 | 0.07 | 0.00 |
|  | *p*-coumaric acid | 0.61 | 17.22 | 0.16 | 4.09 | 1.52 | 0.18 | 0.27 | 0.38 | 3.29 | 0.43 |
|  | coumaric acid | 31.60 | 9.15 | 4.87 | 0.63 | 0.08 | 1.26 | 6.06 | 13.72 | 0.00 | 21.26 |
|  | *t*-cinnamic acid | 6.39 | 0.00 | 0.20 | 0.19 | 0.27 | 1.56 | 2.14 | 0.89 | 0.67 | 0.00 |
|  | caffeic acid | 2.15 | 2.41 | 1.93 | 0.19 | 0.28 | 13.35 | 2.43 | 0.40 | 2.07 | 0.49 |
|  | syringic acid | 0.45 | 10.38 | 0.24 | 1.21 | 1.34 | 4.90 | 4.05 | 4.41 | 1.65 | 0.00 |
|  | galic acid | 3.95 | 0.00 | 0.00 | 0.15 | 0.00 | 0.27 | 0.00 | 0.00 | 0.00 | 0.00 |
|  | sinapic acid | 0.00 | 0.58 | 0.10 | 0.17 | 1.25 | 0.00 | 0.48 | 0.41 | 0.00 | 0.36 |
|  | *p*-hydroxybenzoic acid | 1.10 | 1.21 | 0.00 | 2.36 | 38.26 | 5.99 | 16.21 | 1.70 | 0.90 | 1.35 |
|  | caftaric acid | 0.00 | 0.94 | 2.43 | 3.42 | 2.38 | 4.72 | 6.62 | 3.92 | 3.74 | 1.89 |
|  | 3.4-didydrohyphenolacetic acid | 3.95 | 0.00 | 0.00 | 0.15 | 0.00 | 0.00 | 0.00 | 0.00 | 0.00 | 0.00 |
|  | coutaric acid | 1.39 | 0.36 | 0.37 | 4.29 | 0.33 | 3.46 | 1.16 | 25.23 | 0.53 | 1.40 |
|  | vanillic acid | 0.84 | 5.95 | 0.08 | 2.79 | 0.08 | 2.13 | 6.61 | 5.92 | 1.53 | 0.00 |
| **Flavonoids** | luteolin | 3.95 | 14.67 | 6.48 | 7.52 | 1.01 | 10.17 | 2.36 | 3.41 | 23.31 | 0.13 |
|  | epicatechin | 0.19 | 0.00 | 0.00 | 0.00 | 0.00 | 0.96 | 0.19 | 0.04 | 0.00 | 2.71 |
|  | vitexin | 0.18 | 0.00 | 25.14 | 0.66 | 0.09 | 0.24 | 1.43 | 0.29 | 0.88 | 0.05 |
|  | rutin | 0.81 | 0.09 | 0.46 | 0.07 | 0.00 | 0.00 | 0.00 | 0.09 | 0.00 | 0.23 |
|  | quercetin | 0.30 | 0.56 | 0.20 | 0.20 | 0.98 | 2.50 | 0.66 | 0.29 | 1.62 | 0.24 |
|  | apigenin | 5.29 | 0.00 | 0.09 | 0.94 | 0.15 | 9.34 | 4.69 | 1.74 | 3.01 | 20.98 |
|  | kaempferol | 3.90 | 14.18 | 6.44 | 6.85 | 1.09 | 10.29 | 6.03 | 2.69 | 22.60 | 0.13 |
|  | orientin | 0.00 | 10.25 | 14.75 | 0.19 | 1.03 | 2.12 | 2.73 | 1.82 | 0.38 | 2.33 |
|  | naringenin | 1.29 | 0.91 | 0.25 | 1.01 | 0.62 | 8.98 | 1.02 | 0.00 | 1.08 | 13.54 |
| **Stilbenes** | E-piceid | 0.86 | 0.51 | 0.00 | 0.05 | 0.09 | 0.00 | 0.00 | 0.11 | 0.00 | 0.00 |
|  | Z-piceid | 0.16 | 0.60 | 0.00 | 1.77 | 0.06 | 0.00 | 0.00 | 0.50 | 0.00 | 0.00 |
|  | E-resveratrol | 1.02 | 0.00 | 0.08 | 1.89 | 5.09 | 0.72 | 2.44 | 1.83 | 4.71 | 0.15 |
|  | E-resveratrol | 2.14 | 0.00 | 0.02 | 0.13 | 0.48 | 0.16 | 2.06 | 0.26 | 1.19 | 0.04 |
|  | E-astringin | 0.12 | 0.13 | 0.00 | 0.00 | 0.08 | 0.00 | 0.00 | 0.05 | 0.12 | 21.15 |
|  | Z-astringin | 0.19 | 0.34 | 0.00 | 0.00 | 0.13 | 0.00 | 0.00 | 0.13 | 0.45 | 8.93 |
|  | E-isohapontin | 0.37 | 0.17 | 0.10 | 0.30 | 0.26 | 0.42 | 0.34 | 0.63 | 0.40 | 0.13 |
|  | ampelopepsin A | 2.46 | 0.61 | 0.21 | 0.13 | 0.00 | 0.24 | 0.00 | 0.00 | 0.67 | 0.00 |
| Sum of phenolic compounds [mg/g]^*^ | | 0.33 ± 0.00^c^ | 0.91 ± 0.00^a^ | 0.14 ± 0.00^d^ | 0.04 ± 0.00^g^ | 0.05 ± 0.00^f^ | 0.02 ± 0.00^h,i^ | 0.02 ± 0.01^i^ | 0.47 ± 0.01^b^ | 0.02 ± 0.00^g,h^ | 0.08 ± 0.00^e^ |

* Data are expressed as mean ± SD (n = 3). Means in line related to the sum of phenolic compounds for each herb followed by the different letters are significantly different (P < 0.05).


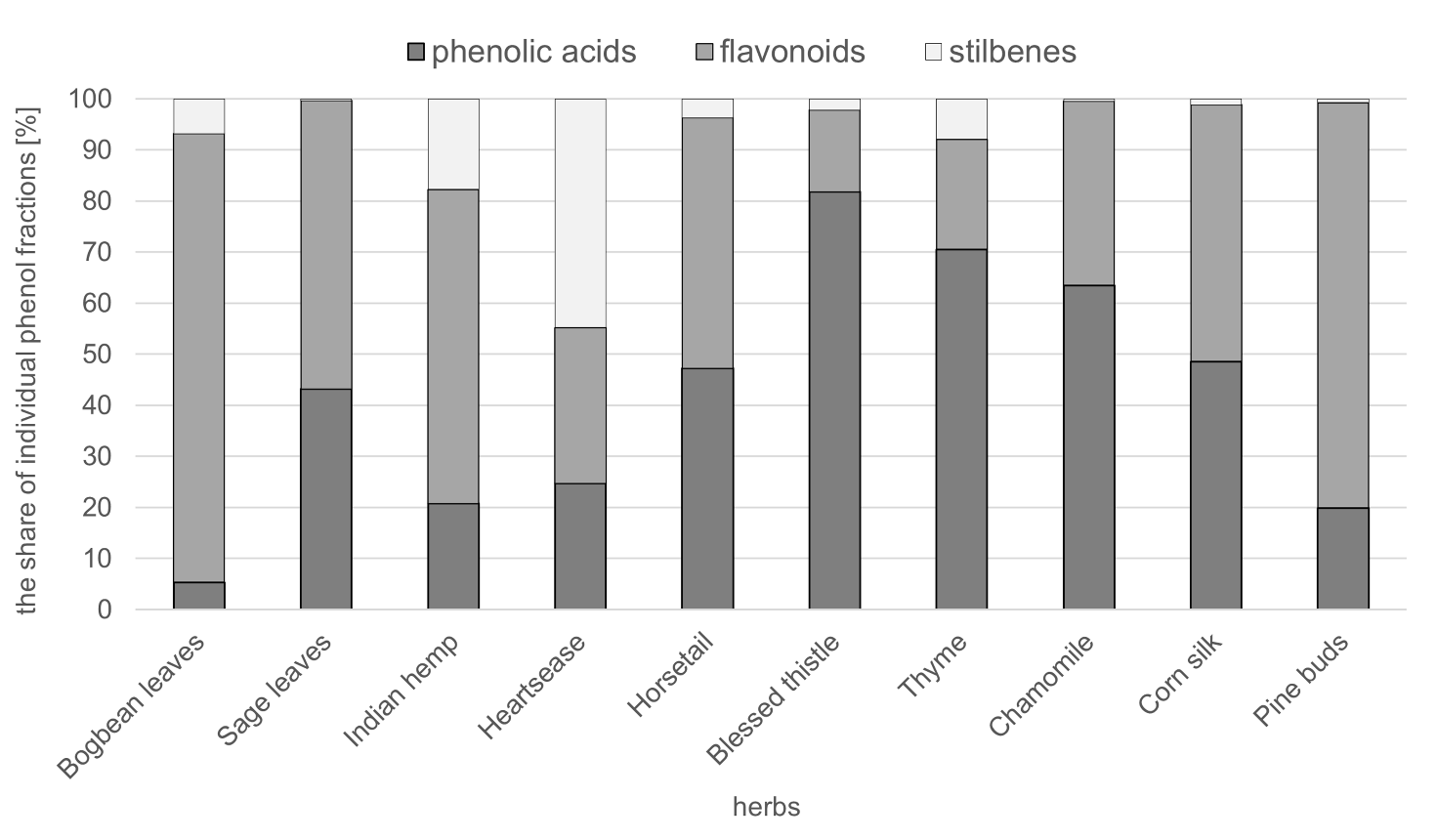


Fig S1. Contribution of particular phenolic compounds group in the non-hydrolyzed extracts.


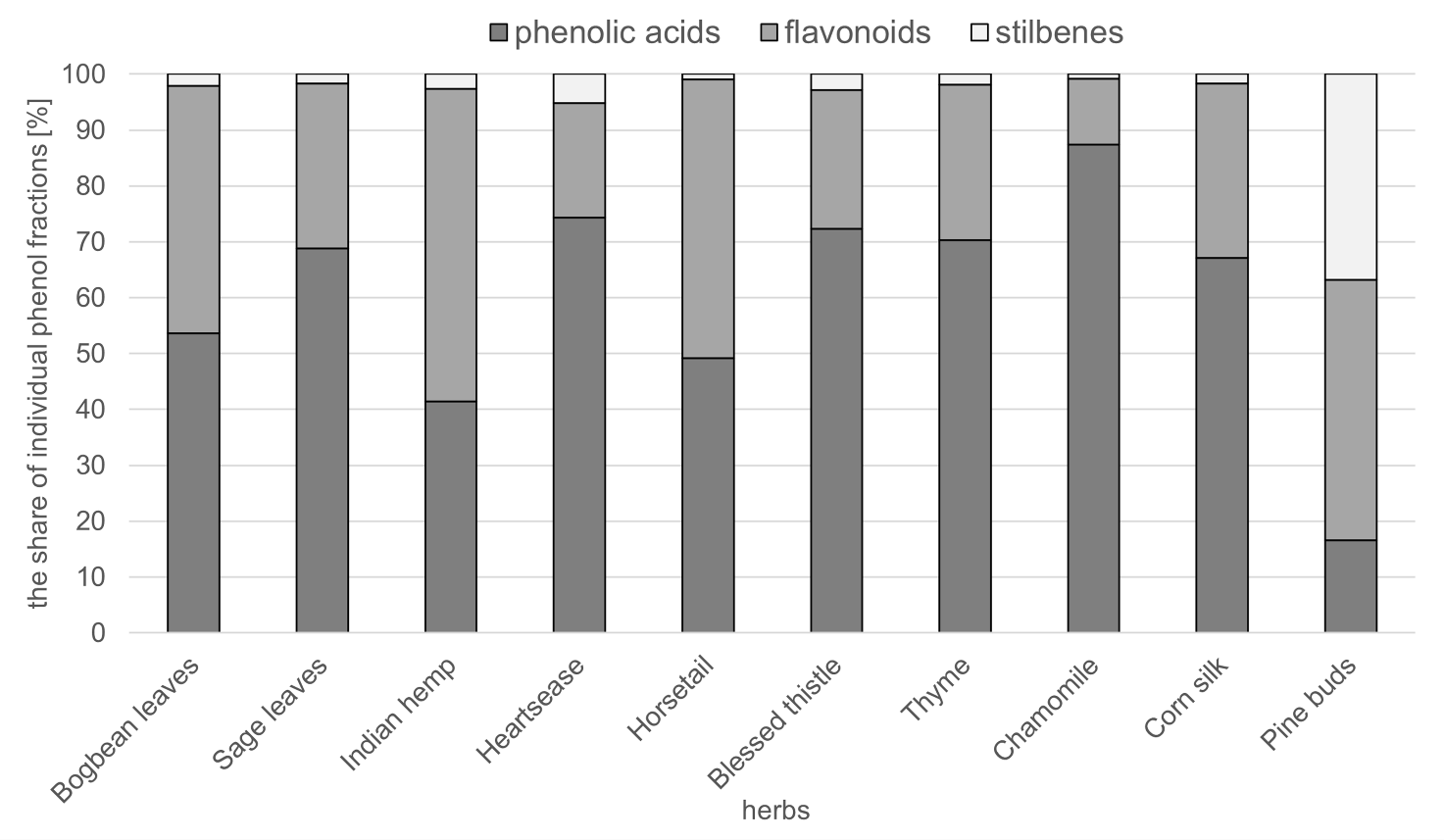


Fig S2. Contribution of particular phenolic compounds group in the extracts after alkaline hydrolysis (phenolic compounds released from ester derivatives).


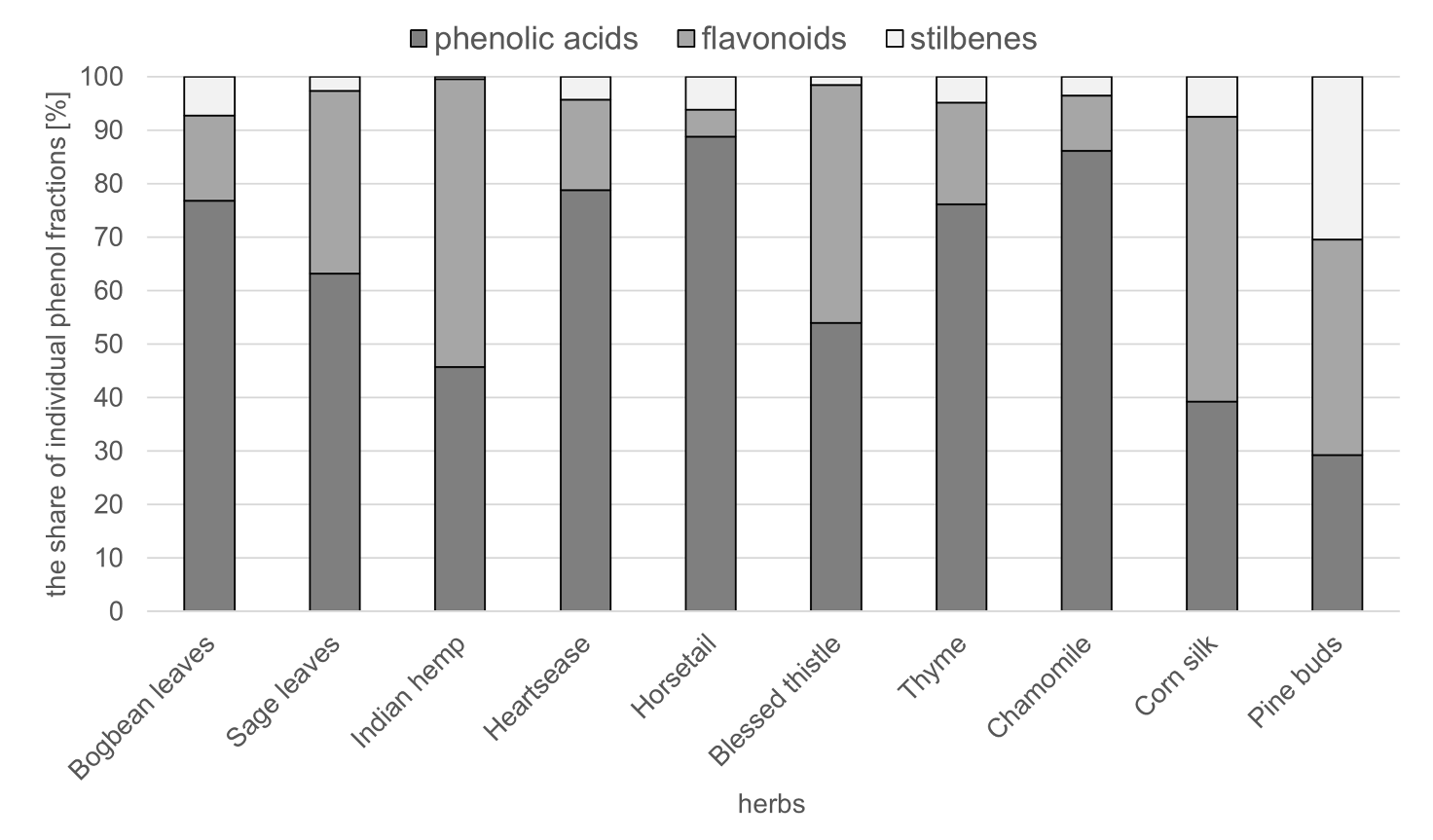


Fig S3. Contribution of particular phenolic compounds group in the extracts after acid hydrolysis (phenolic compounds released from glycosidic derivatives).
